# Sialic acids regulate microvessel permeability, revealed by novel *in vivo* studies of endothelial glycocalyx structure and function

**DOI:** 10.1113/JP274167

**Published:** 2017-08-01

**Authors:** Kai B. Betteridge, Kenton P. Arkill, Christopher R. Neal, Steven J. Harper, Rebecca R. Foster, Simon C. Satchell, David O. Bates, Andrew H. J. Salmon

**Affiliations:** ^1^ Bristol Renal, Schools of Clinical Sciences and Physiology & Pharmacology, Dorothy Hodgkin Building University of Bristol Bristol BS1 3NY UK; ^2^ School of Medicine, Faculty of Medicine and Health Sciences University of Nottingham Medical School Nottingham NG7 2UH UK; ^3^ Biofisika Institute (CSIC UPV/EHU) and Research Centre for Experimental Marine Biology and Biotechnology (PiE) University of the Basque Country Spain; ^4^ Renal Service, Specialist Medicine and Health of Older People Waitemata DHB Auckland New Zealand

**Keywords:** Permeability, Sialic acid, glycocalyx, Correlative Light and Electron Microscopy

## Abstract

**Key points:**

- We have developed novel techniques for paired, direct, real‐time *in vivo* quantification of endothelial glycocalyx structure and associated microvessel permeability.
- Commonly used imaging and analysis techniques yield measurements of endothelial glycocalyx depth that vary by over an order of magnitude within the same vessel.
- The anatomical distance between maximal glycocalyx label and maximal endothelial cell plasma membrane label provides the most sensitive and reliable measure of endothelial glycocalyx depth.
- Sialic acid residues of the endothelial glycocalyx regulate glycocalyx structure and microvessel permeability to both water and albumin.

**Abstract:**

The endothelial glycocalyx forms a continuous coat over the luminal surface of all vessels, and regulates multiple vascular functions. The contribution of individual components of the endothelial glycocalyx to one critical vascular function, microvascular permeability, remains unclear. We developed novel, real‐time, paired methodologies to study the contribution of sialic acids within the endothelial glycocalyx to the structural and functional permeability properties of the same microvessel *in vivo*. Single perfused rat mesenteric microvessels were perfused with fluorescent endothelial cell membrane and glycocalyx labels, and imaged with confocal microscopy. A broad range of glycocalyx depth measurements (0.17–3.02 μm) were obtained with different labels, imaging techniques and analysis methods. The distance between peak cell membrane and peak glycocalyx label provided the most reliable measure of endothelial glycocalyx anatomy, correlating with paired, numerically smaller values of endothelial glycocalyx depth (0.078 ± 0.016 μm) from electron micrographs of the same portion of the same vessel. Disruption of sialic acid residues within the endothelial glycocalyx using neuraminidase perfusion decreased endothelial glycocalyx depth and increased apparent solute permeability to albumin in the same vessels in a time‐dependent manner, with changes in all three true vessel wall permeability coefficients (hydraulic conductivity, reflection coefficient and diffusive solute permeability). These novel technologies expand the range of techniques that permit direct studies of the structure of the endothelial glycocalyx and dependent microvascular functions *in vivo*, and demonstrate that sialic acid residues within the endothelial glycocalyx are critical regulators of microvascular permeability to both water and albumin.

## Key points

- We have developed novel techniques for paired, direct, real‐time *in vivo* quantification of endothelial glycocalyx structure and associated microvessel permeability.
- Commonly used imaging and analysis techniques yield measurements of endothelial glycocalyx depth that vary by over an order of magnitude within the same vessel.
- The anatomical distance between maximal glycocalyx label and maximal endothelial cell plasma membrane label provides the most sensitive and reliable measure of endothelial glycocalyx depth.
- Sialic acid residues of the endothelial glycocalyx regulate glycocalyx structure and microvessel permeability to both water and albumin.

**Abbreviations**
Alexa 488: Alexa Fluor 488
BSA: bovine serum albumin
FITC: fluorescein isothiocyanate
FWHM: full width at half‐maximum
*P*_sBSA_: apparent solute permeability to bovine serum albumin
R18: octadecyl rhodamine B chloride
S1P: sphingosine‐1‐phosphate
TMA‐DPH: 1‐(4‐trimethylammoniophenyl)‐6‐phenyl‐1,3,5‐hexatriene *p*‐toluenesulfonate
TRITC: trimethylrhodamineisothiocyanate
WGA: wheat germ agglutinin

## Introduction

The entire luminal surface of all vascular walls is covered with a continuous, carbohydrate‐rich mesh: the endothelial glycocalyx (Weinbaum *et al*. 2007). The membrane‐bound portion of the endothelial glycocalyx is supplemented with components of plasma to form a thickened endothelial surface layer. This endothelial coat governs the nature of interactions between fluid, protein and cellular constituents of flowing blood and the endothelial cell, and is therefore a critical regulator of vascular function (Curry & Adamson, 2013). The endothelial glycocalyx regulates permeability in exchange microvessels; leukocyte rolling, adhesion and migration in post‐capillary venules; mechanotransduction, flow‐ and shear‐induced vasodilatation in arterioles; and protects the vessel wall from the development of atherosclerosis in conduit arteries.

The role of the endothelial glycocalyx in regulating microvessel permeability was formalised in the fibre‐matrix junction‐break theory proposed by Curry and Michel in 1980. This theory and subsequent refinements hold that the endothelial glycocalyx is the primary macromolecular sieve in continuous microvessels, and that this sieving immediately above interendothelial junctions creates a protected ‘sub‐glycocalyx space’, whose oncotic pressure determines the magnitude of the oncotic pressure gradient resisting water and solute flux across vessel walls (Levick & Michel, 2010). Demonstrations of the resulting non‐linear form of the Starling equation under steady‐state filtration conditions (Michel & Phillips, 1987), and unequal actions of luminal and abluminal oncotic pressures (Hu *et al*. 2000), support this theory. These functional demonstrations are supported by recent observations that a repeating periodic arrangement of the fibres of the endothelial glycocalyx are sufficient to explain the near‐uniform macromolecular permeability coefficients seen throughout the vasculature (Squire *et al*. 2001; Arkill *et al*. 2011). The endothelial glycocalyx also regulates hydraulic resistance, with greater glycocalyx depths providing greater resistance (Adamson, 1990; Salmon *et al*. 2009).

The structure of the endothelial glycocalyx is complex, with depth, spatial localisation and 3‐D architecture all structural facets that impact on vascular function, including permeability. In addition, determining the relative functional importance of different glycocalyx components has been challenging. The endothelial glycocalyx contains an array of glycoproteins, proteoglycans and glycosaminoglycans (GAGs), with much focus on the importance of GAGs in regulating key physiological properties to the vessel wall (Tarbell & Ebong, 2008). Many of the glycoproteins are capped by sialic acid (Varki, 2008) contributing to the structure of the glycocalyx. The dominant GAG is heparan sulphate, which is a family of charged molecules with a wide variety of subtypes depending on the nature of bonds between individual saccharide units and their sulphation pattern (Weinbaum *et al*. 2007). Heparan sulphate has been shown to regulate mechanotransduction (Florian, 2003; Kumagai *et al*. 2009), and evidence indicates an important role in regulating permeability in endothelial monolayers *in vitro* (Singh *et al*. 2007) and in isolated coronary arterioles *ex vivo* (Huxley & Williams, 2000). Chondroitin sulphate also carries significant charge, and again appears to have an important role in regulating permeability (Jeansson & Haraldsson, 2003). Finally hyaluronan, a non‐sulphated GAG with extended chain length, also plays important roles in mechanotransduction and glomerular filtration (Jeansson *et al*. 2006, 2009; Pahakis *et al*. 2007).

At present, little is known about the potential role that sialic acid residues within the endothelial glycocalyx may also make to microvessel permeability characteristics. Sialic acid residues regulate leukocyte (Sakarya *et al*. 2004) and platelet (Gorog *et al*. 1982) adhesion, mechanotransduction (Tarbell & Ebong, 2008; Kumagai *et al*. 2009), low‐density lipoprotein (LDL) uptake into endothelial cells (Gorog *et al*. 1982) and development of atherosclerosis (Cuniberti *et al*. 2005). Endothelial glycocalyx sialic acid residues are disrupted in disease states predisposing to atherosclerosis such as diabetes (Nassimizadeh *et al*. 2010), as well as playing an important role in kidney disease (Dekan *et al*. 1991; Gelberg *et al*. 1996), and sepsis (Adembri *et al*. 2011), all of which have been linked to endothelial glycocalyx dysfunction (Salmon & Satchell, 2012). Despite this, the role of sialic acids in regulating microvessel permeability remains unclear. Early studies showed no change in microvessel permeability following sialic acid disruption by neuraminidase (Mason *et al*. 1977). *In vitro* studies indicate a role for sialic acid residues in endothelial cell monolayer barrier function (Singh *et al*. 2007; Cioffi *et al*. 2012), but the endothelial glycocalyx and permeability coefficients of cultured endothelial cells are substantially different from the *in vivo* situation (Curry, 2005; Chappell *et al*. 2009). Despite their abundance and importance in key physiological processes and disease states, the importance of endothelial glycocalyx sialic acid residues in regulating intact vessel wall permeability coefficients remains unclear.

Current *in vivo* studies of the endothelial glycocalyx either involve disruptive (e.g. electron microscopy) or indirect (e.g. tracer exclusion) techniques to examine the endothelial glycocalyx, both of which give conflicting results (Salmon *et al*. 2009; Gao & Lipowsky, 2010). Newer and more direct optical imaging techniques which have employed the use of fluorescently tagged antibodies and lectins to bind glycocalyx have also been attempted in recent years (Cai *et al*. 2012; Salmon *et al*. 2012; Yen *et al*. 2012). These techniques have often used confocal laser scanning and two‐photon microscopes in an attempt to better resolve the glycocalyx in microvessels. However, even with the improved resolution of laser scanning methods, these optical techniques are still assessing changes in glycocalyx structure that fall within or below the diffraction limit of optical imaging, particularly in the physiological environment (Gretz & Duling, 1995). To make estimates of relative changes in depth within this resolution limit, each of these direct optical imaging techniques have applied different analysis methods which often yield varying and conflicting results (Gao & Lipowsky, 2010; Cai *et al*. 2012; Salmon *et al*. 2012; Yen *et al*. 2012). In addition, these studies of endothelial glycocalyx are rarely coupled with measurements of the functional properties of the wall of the same vessel.

The aim of this study was to understand the contribution of endothelial glycocalyx sialic acid residues to microvascular structure and permeability function, and to do so we have developed methods for real‐time, direct, paired measurements of endothelial glycocalyx structure and function *in vivo*, as well as applying electron microscopy preservation to compare *in vivo* and *ex vivo* measurements of the same vessels with known functional properties.

## Methods

### Ethical approval

Procedures were carried out in accordance with national regulations set out by the UK Home Office legislation, and with local approval from the University of Bristol local ethical committee. Male Sprague–Dawley rats (150–250 g; Harlan, Oxon, UK) were used in all experiments. Anaesthesia was induced with an intraperitoneal injection of sodium pentobarbital (60 mg kg^−1^), and maintained with subcutaneous intramuscular injections approximately every 45 min. At the end of all experiments animals were killed with excess anaesthetic and cervical dislocation. Glycocalyx measurements were made on 16 rats, solute permeability measurements on 9 rats, and hydraulic conductivity and reflection coefficient measurements on 7 rats.

### Real‐time endothelial glycocalyx imaging

Under anaesthesia, the mesentery was exposed via a midline laparotomy, constantly superfused with mammalian Ringer solution at 37°C, teased over a glass coverslip, and imaged via the objective of an inverted microscope. Capillaries and post‐capillary venules from 10 to 40 μm in diameter and without rolling or adherent leukocytes were identified and cannulated in the direction of flow with a refillable bevelled glass micropipette (Bates *et al*. 2002) for endothelial glycocalyx imaging alone in initial experiments. Microvessels were then perfused continuously with mammalian Ringer solution containing bovine serum albumin (BSA; 4%; A4378, Sigma, Dorset, UK), followed by supplementation with an endothelial cell membrane label (octadecyl rhodamine B chloride (R18); 2 μm in BSA–Ringer–0.01% DMSO; O246, Life Technologies, Renfrew, UK), or TMA‐DPH (1‐(4‐trimethylammoniophenyl)‐6‐phenyl‐1,3,5‐hexatriene *p*‐toluenesulfonate; 2 μm in BSA–Ringer–0.01% DMSO; T204, Molecular Probes, Eugene, OR, USA) (Peti‐Peterdi *et al*. 2009) and then labelling of sialic acid residues within the endothelial glycocalyx (fluorescein isothiocyanate (FITC)‐conjugated wheat germ agglutinin (WGA; 2 mol mol^−1^ dye content; 2.77 μm in BSA–Ringer; L4895, Sigma) or trimethylrhodamineisothiocyanate (TRITC)‐conjugated WGA (0.65 mol mol^−1^ dye content; 11.08 μm in BSA–Ringer; L5266, Sigma), with concentrations determined in preliminary optimisation experiments, each for 5 min, followed by washout with unsupplemented 4% BSA–Ringer solution.

Respiratory and peristaltic movement required both fluorophores (R18 and FITC‐WGA) to be excited and detected simultaneously in all experiments. The consequent theoretical possibility of signal detection in the 605 ± 75 nm (R18) channel from the broad emission spectrum of WGA‐conjugated FITC was assessed qualitatively and quantitatively. Fluorescein (FITC‐WGA) artefacts observed in the centre of vessels were detected in both the 515 ± 30 nm (FITC) and 605 ± 75 nm (R18) channels. Due to simultaneous dual colour fluorescence collection overlap of the R18 emission with the 488 laser can be significant therefore other fluorophores used in combined glycocalyx–permeability studies (FITC‐WGA and Alexa Fluor 488 (Alexa 488) BSA) also required assessment of alternative glycocalyx and endothelial cell membrane labels. Endothelial glycocalyx depth was therefore also measured using TRITC‐WGA (to label sialic acid residues in the endothelial glycocalyx), and TMA‐DPH (to label endothelial cell membrane), with neither fluorophore emission spectra detectable by an alternative channel. This combination of TMA‐DPH (endothelial cell membrane) and TRITC‐WGA (sialic acid residues in the endothelial glycocalyx) also eliminated the possibility of fluorescence emission from anatomical labels from confounding the functional solute flux data obtained using Alexa 488 BSA in later experiments.

**Figure 1:**
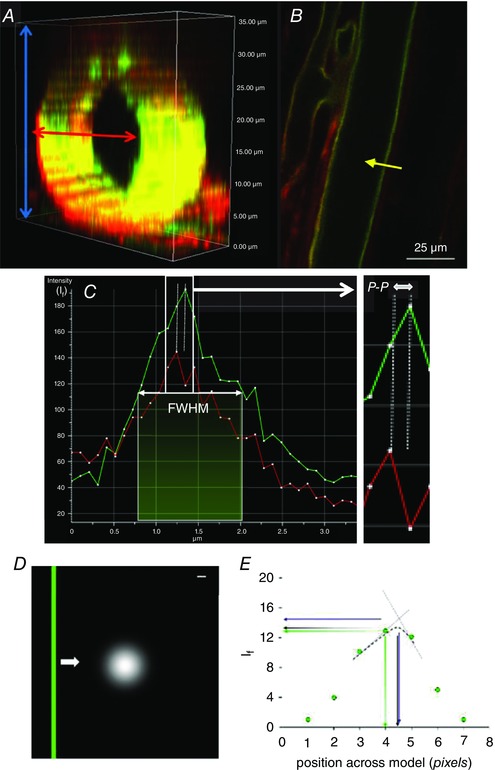
Imaging and analysis of endothelial glycocalyx *in vivo* *A*, section of a *z*‐stack imaged *in vivo*; when a measurement line was drawn across the width of a vessel, all available slices (blue arrows) were scanned though and the slice which yielded the widest vessel diameter (red arrow) was selected for measurements. *B*, example of FITC‐WGA (green) and R18 (red) imaged vessel in the mid‐plane region. For demonstration purposes the intensity profile (yellow arrow) has only been drawn on one side of the vessel; this line was actually drawn across the entire width and therefore took measurements of both sides simultaneously. *C*, the intensity profile of both R18 and FITC‐WGA along a line across the vessel wall (yellow arrow) was used to determine 4 parameters of glycocalyx depth: (1) the anatomical distance between peak signals from R18 and FITC‐WGA labels (peak to peak: P‐P) as a measure of depth, (2) the full width measured at the point of half‐maximal fluorescence intensity of FITC‐WGA (FWHM) and (3) the peak intensity of FITC‐WGA (*I*_f_), and (4) fractional coverage of endothelial glycocalyx was calculated as the number of points with a peak to peak endothelial glycocalyx depth measurement >0. *D*, a model of endothelial glycocalyx (green bar) was moved (white arrow) at 1 nm intervals across an Airy disc representation of laser light (white circle). Scale bar: 100 nm. *E*, the fluorescence intensity (*I*_f_) of measurements across the green bar (green points) was highest when peak *I*_f_ was calculated by triangulation (interrupted straight blue lines, blue arrows), lowest from absolute values (green points and arrows), and intermediate when peak *I*_f_ was calculated from a Gaussian distributions (interrupted black curve, black arrows).

Labelled mesenteric microvessels were imaged via the objective (CFI Plan Apochromatic VC 60× Oil with 1.4 NA) of an inverted microscope (Nikon Eclipse TiE), and scanned in the *x* and *y* directions using 488 nm and 543.3 nm wavelength lasers (100% laser power). The laser power, gain and offset for both channels was kept constant for all experiments (515 ± 30 nm channel = 90 gain and 0 offset; 605 ± 75 nm = 100 gain and 0 offset). Fluorescent light emitted back into the objective was detected using 515 ± 30 nm and 605 ± 75 nm photomultiplier tubes (PMTs), and differential interference contrast (DIC) images were detected simultaneously in a diascopic manner using a PMT for transmitted light. *z*‐stack images (2048 by 2048 pixels; 20 slices) were captured through the entire vessel using an even inter‐slice distance in proportion to the height of the vessel being imaged (e.g. a 20 μm diameter vessel will have 20 optical slices each 1 μm apart) (Fig. 1 *A*).

### Endothelial glycocalyx image analysis

All *z*‐stacks were analysed using FIJI (Image J) software (Schindelin *et al*. 2012). Three channels (DIC, endothelial glycocalyx label (FITC or TRITC), and cell membrane label (R18 or TMA‐DPH channels)) and 20 slices in the *z* plane were processed to account for variability in the image acquisition step, predominantly arising from respiratory and peristaltic movement between optical slices of the *z*‐stacks causing misalignment of frames within a *z*‐stack. Virtual stack slices were therefore registered based on the images acquired in the DIC channel, and realigned identically for all channels to generate a 3‐D image of the entire vessel. Once registered, the *z*‐stack image demonstrating the maximal vessel diameter (i.e. the mid‐point of the vessel, and the position in which endothelial glycocalyx and cell membrane labels will be perpendicular to the plane of section, minimising anatomical overlap) was identified (Fig. 1 *A*, red arrow). Fluorescence intensity profiles were then generated for both endothelial cell label and the sialic acid component of the endothelial glycocalyx label, along 30 evenly‐spaced lines across the full width of the vessel. Each point along the profile represented the average fluorescence intensity from 16 adjacent pixels (8 pixels either side of the line drawn) (Fig. 1 *B*). Four automated measurements of endothelial glycocalyx parameters were then calculated (Fig. 1 *C*): (i) the anatomical distance between peak signal intensity of the endothelial cell membrane label and sialic acid component of the endothelial glycocalyx label (‘peak to peak’); (ii) the full width measured at the point of half‐maximal fluorescence intensity of the sialic acid component of the endothelial glycocalyx label (‘FWHM’); (iii) the peak fluorescence intensity of the sialic acid component of the endothelial glycocalyx label (*I*_f_); and (iv) fractional coverage of positively (and negatively) resolvable glycocalyx. Mean values for each of the first three assessments of the endothelial glycocalyx were determined using data from all of the 30 intensity lines analysed; any values for peak to peak which yielded depth values of ≤0 were excluded from the calculation of the mean glycocalyx depth and were used to determine (iv). All registration, maximal width identification, fluorescence intensity profiling and measurement steps were conducted by automated macros.

To model whether the peak to peak method was dependent on the size of the individual picture elements acquired in the image we used computational modelling to vary the anatomical position of the ‘true’ theoretical peak glycocalyx within a single voxel by passing an Airy disc representation of 488 nm laser light passing through a 1.4 NA objective (Fig. 1 *D*) at 1 nm intervals. The anatomical position of the maximum intensity reading would vary as much as one voxel integer (104 nm for the 60× objective). This problem was addressed mathematically by comparing the recorded peak intensity with those calculated to be close to the true intensity via linear interpolation (triangulation) and Gaussian fitting of the three peak pixels recorded (Fig. 1 *E*). Results showed that recorded peak intensities and triangulated peak intensities varied depending on the true position, but Gaussian intensities provided the least variable (mid‐range) results, with the position of peak fluorescence intensity identified independent of the starting point of the scan, therefore providing the greatest anatomical resolution of these three methods. (Fig. 1 *E*). As a consequence, all further measurements were calculated using the peak value of a Gaussian fit of recorded peak intensities.

### Paired imaging of solute flux and endothelial glycocalyx

**Figure 2:**
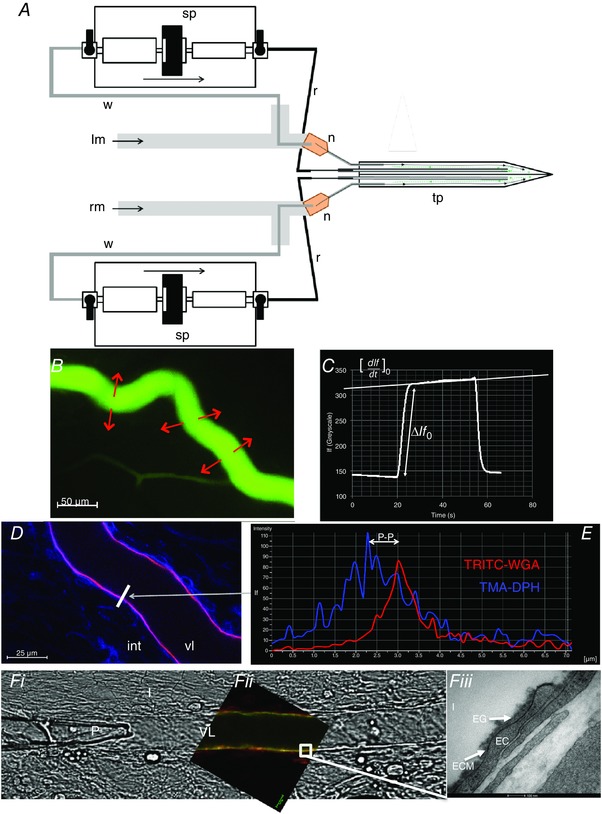
Paired imaging of solute permeability and endothelial glycocalyx in single microvessels *A*, two 26G needles (n) and two fine tubes (grey line) were inserted into each of the main barrels of the bevelled theta‐style micropipette (tp) and glued in place to form a tight seal. Two syringe pumps (sp) were used to push solution into the fine tube which was connected to the pump by a refill line (r) and both sides filled with BSA–Ringer solution (or desired dye). The other end of the syringe pumps (sp) were connected to a waste line (w) that was positioned directly behind the back of the two needles (n) and would pull solution away (green arrows) as the refill line pushed solution into (black arrows) the pipette. The needles were also connected to two separate water manometers; the right manometer (rm) and the left manometer (lm) to control pressure on each side of the pipette independently. This design allowed the solution in the pipette to be readily changed even during microvessel cannulation. *B*, mesenteric microvessels of pentobarbitone‐anaesthetised male Sprague–Dawley rats were cannulated using theta‐style micropipettes where the pressure on either side was set so that only one desired side perfused (15–30 cmH_2_O). Perfused BSA–Ringer solution was switched instantly to perfused Alexa 488‐labelled BSA (0.03 mg ml^−1^) transiently for 30–60 s, and imaged using fluorescence microscopy. *C*, a measuring window is drawn across the vessel and areas surrounding it and the change in mean fluorescence intensity (*I*_f_) is plotted against time (*t*). Initial filling of the vessel with Alexa 488‐labelled BSA causes a stepwise increase in *I*_f_ (Δ*I*_f0_), followed by a steady linear increase in *I*_f_, the initial rate of change of which ([d*I*_f_/d*t*]_0_) represents the rate of solute flux across the vessel wall. *D*, confocal image of cannulated mesenteric microvessel perfused and labelled with TMA‐DPH (endothelial cell membrane: blue) and TRITC‐WGA (sialic acids within endothelial glycocalyx: red). vl: vessel lumen; int: interstitium. White bar: selected line of interest across vessel wall. *E*, fluorescence intensity profile along selected white bar. P‐P (peak to peak): measure of anatomical distance between peak fluorescence of TMA‐DPH (endothelial cell membrane: blue) and TRITC‐WGA (sialic acids within endothelial glycocalyx: red) signals. FWHM: measure of full‐width half‐maximum of TRITC‐WGA (sialic acids within endothelial glycocalyx: red) signal. Other previously described parameters were also analysed (mean peak *I*_f_ of TRITC‐WGA, and fractional coverage). *F*, representative images of the same cannulated and perfused microvessel imaged using light (*a*), confocal (*b*) and electron (*c*) microscopy. *Fa*, P: pipette; I: interstitium; VL: vessel lumen. *Fb*, red: endothelial cell membrane (R18); green: endothelial glycocalyx (FITC‐WGA); white box: portion of membrane also examined by electron microscopy. *Fc*, VL: vessel lumen; EG: endothelial glycocalyx; ECM: endothelial cell membrane; EC: endothelial cell; I: interstitium.

Having developed imaging methods for studying the endothelial glycocalyx in real time *in vivo*, alternative cannulation and perfusion systems were necessary and hence developed for combined solute permeability and endothelial glycocalyx imaging (Fig. 2 *A*). Micropipettes were constructed in‐house from split‐barrel borosilicate glass capillaries (o.d. 2 mm, wall thickness 0.3 mm, central septum thickness 0.22 mm), pulled to a fine tip (Narishige P22), bevelled (25 deg angle, 10–15 μm tip diameter), and angled mid‐shaft (∼45 deg angle). Each pipette barrel then received a refilling line (stretched polyethylene tubing, o.d. 0.61 mm) and a blunt‐tipped needle (26G), before the non‐cannulating rear aperture was sealed with epoxy resin. The refill line was connected to a syringe pump‐driven replaceable refill reservoir for exchanging solutions within each pipette barrel; the 26G needles were attached to individual, modifiable water manometers to control hydrostatic pressures in each side of the pipette independently (Fig. 2 *A*).

Using the same surgical preparation described above for glycocalyx‐only imaging, mesenteric microvessels were cannulated using this double‐barrelled pipette system. Barrel A contained BSA–Ringer solution (final concentration 4% w/v BSA). Barrel B contained BSA–Ringer solution supplemented with Alexa Fluor 488‐conjugated BSA (Alexa 488 BSA; 0.03 mg ml^−1^ unless otherwise stated) to a final concentration of 4% w/v total BSA. Following cannulation, the pressures exerted individually to both pipette barrels were adjusted manually using the relevant water manometers until balance pressure was reached (perfusion ceased; typical range 15–25 cmH_2_O). The pressure in barrel A (BSA–Ringer) was then marginally raised (1–5 cmH_2_O) above the pressure in barrel B (Alexa 488 BSA) until the vessel was perfused with BSA–Ringer solution only, without backflow into barrel B. The vessel was continuously perfused under these stable conditions for 10 min before image acquisition.

The entire imaging window was excited at 480 nm using a widefield fluorescent light‐emitting diode (LED) (Precise Excite Cool LED; 50% power). Emitted fluorescence was filtered through a 535 nm filter block and focused onto the sensor of a high sensitivity camera (Photometrics CoolSnap HQ2; fixed settings: 14 bit monochrome detection; 20 MHz readout mode; 2 × 2 binning; 696 × 520 pixels; 350 visual gain). Under these conditions, initial videos were captured (Nikon NIS Elements software; 60 ms intervals; 20 s duration). Pressures in barrels A and B were then instantly swapped, halting perfusion of BSA–Ringer solution and commencing perfusion with Alexa 488 BSA (Fig. 2 *B* and *C*). After 20 s perfusion with fluorescent solution, pressures were switched back to initial settings, generating perfusion with non‐fluorescent BSA–Ringer for a final period of 20 s (Fig. 2 *C*). This perfusion and imaging sequence for capturing solute permeability (*P*_sBSA_) quantification data was repeated 4–5 times for each period of the experiment. During these experimental periods, perfusate either contained vehicle, or neuraminidase to disrupt sialic acids (neuraminidase from *Clostridium perfringens*, Salmon *et al*. 2012; 2 U ml^−1^ in 4% BSA; N2876, Sigma).

Following this imaging of solute flux, pipette barrels A and B were refilled and the vessel perfused with labels for endothelial cell membrane and glycocalyx: note that the use of Alexa 488‐labelled BSA precluded the use of overlapping emission fluorophores (e.g. FITC) for cell membrane or glycocalyx labelling in these permeability experiments. After *P*_sBSA_ measurements, vessels were therefore labelled with TMA‐DPH to label endothelial cell membranes; and TRITC‐WGA to label endothelial glycocalyx sialic acid residues; Fig. 2 *D*). Each dye was perfused for 5 min, before lumen refill and perfusion with BSA–Ringer solution alone. Endothelial glycocalyx was then imaged as described above (Fig. 2 *E*).

### Solute permeability analysis

Real‐time video images of Alexa 488 BSA‐perfused microvessels were analysed using Nikon NIS Elements software. A standard region of interest (ROI) was created covering the full length of the perfused vessel in the imaging field and extending 20 μm directly adjacent to either side of the vessel, with the distance between pipette tip and the ROI position maintained at ∼150 μm. ROI measurements of mean fluorescence intensity (*I*_f_) *vs*. time (seconds) were recorded from each experiment and plotted as a scatter plot giving a characteristic time course appearance (e.g. Fig. 2 *C*). Anatomical dimensions of the microvessel radii (*r*) were measured directly from images of endothelial cell membrane labelling in the same microvessel.

A typical time course plot (e.g. Fig. 2 *C*) demonstrated a steady baseline value of background fluorescence intensity (*I*_f_) during perfusion of BSA–Ringer alone. Initial filling of the vessel lumen with Alexa 488 BSA caused a sharp, stepwise increase in fluorescence intensity (*I*_f_; [Δ*I*_f_]_0_)). Initial BSA transport across the vessel wall caused a steady rate increase in *I*_f_ over time ([d*I*_f_/d*t*]_0_) (Fig. 2 *C*; white line), followed by a sudden drop in *I*_f_ when the perfusate was switched back to BSA–Ringer solution.

The permeability coefficient of the solute BSA (*P*_sBSA_) was the rate of flux of BSA (*J*_sBSA_) through an area of a microvessel (*A*) per unit concentration difference (Δ*c*). This can be expressed as:

(1) P sBSA =J sBSA A×1Δc

With this methodology (Huxley *et al*. 1987), *J*_sBSA_ could be calculated from the initial stepwise change (Δ*I*_f_) and subsequent rate of change in fluorescence intensity (d*I*_f_) over time (*t*) immediately (time zero: ‘0’) after the capillary lumen was filled with Alexa 488 BSA in a capillary of measured radius (*r*):

(2) P sBSA =1ΔIf0×dIfdt×r2

### Endothelial glycocalyx imaging by electron microscopy

Following real‐time structural measurements of glycocalyx and vessel permeability *in vivo*, vessels were then perfused with albumin‐free Alcian Blue‐Ringer solution (Alcian Blue 8GX, A5268, Sigma) as previously described (Salmon *et al*. 2009) for 5 min (Fig. 2 *F*). Superfusate was then switched from warm Ringer solution to ice‐cold glutaraldehyde (2.5%) for 5 min to achieve mesentery stiffening indicating successful fixation. Micropipettes were then removed, the mesenteric panel containing the preserved vessel dissected away from the surrounding gut, rinsed and then stored in glutaraldehyde at 4°C. Specimens were post fixed using 1% osmium tetroxide, 1% lanthanum chloride and saturated uranyl acetate on ice, and then processed and embedded into resin. The cannulation site in the microvessel studied *in vivo* was identified, and the block was trimmed until 100 nm sections containing the previously studied portion of mesenteric microvessel were cut, mounted on piloform covered slot grids, and imaged with 120 keV transmission electron microscopy (Tecnai T12 with an Eagle camera (FEI)) (Fig. 2 *Fc*).

Electron micrographs were analysed using Adobe Photoshop software. Images from eight points evenly spaced around the lumen were assessed to quantify glycocalyx depth in each of these regions. The depth was assessed at 20 points for each high power image by measuring the anatomical distance from the luminal phospholipid bilayer to the furthest point of the glycocalyx; perpendicular measurements were ensured by only using images where the phospholipid bilayer was visible as two distinct layers. This was performed by initially overlaying a grid, and depth was measured at any point where the visible phospholipid bilayer crossed vertical or horizontal lines (depending on the orientation of the image). Sample groups were blinded before analysis, and measurements performed at points where endothelial cell membranes touched the overlaid gridlines to eliminate potential subjective interpretations.

### Hydraulic conductivity and reflection coefficient measurement

In separate experiments, we used the Landis–Michel micro‐occlusion technique as previously described to measure permeability coefficients hydraulic conductivity (*L*_p_) and the reflection coefficient to albumin (σ_BSA_) in intact microvessels *in vivo* (Salmon *et al*. 2009).

In the steady state the following holds (Michel & Phillips, 1987):

(3) $$\frac{J_{v}}{A}=\;L_{p}\left(\Delta P-\;\sigma^{2}\Pi_{c}\right)$$

Individual mesenteric microvessels were exposed, cannulated with a single‐lumen bevelled micropipette, and perfused with 4% BSA–Ringer solution supplemented with low‐density rat erythrocytes (as flow markers and source of sphingosine‐1‐phosphate (S1P)) at 60, 80 or 100 cmH_2_O. Vessels were intermittently and briefly occluded with a fine glass rod. Fluid filtration rate per unit area (*J*_v_/*A*) was calculated from the rate of erythrocyte movement towards the occluder and the cross‐sectional area of the vessel. *L*_p_ (×10^−7^ cm s^−1^ cmH_2_O^−1^) was calculated from the slope of the relationship between filtration rate and applied pressure, and the measured effective oncotic pressure difference (σΔπ) from the abscissal intercept. The ideal oncotic pressure of this perfusate (π*_c_*) was calculated empirically (Bates, 1998). The reflection coefficient was subsequently calculated from the relation (Michel & Phillips, 1987):

(4) $$\sigma =\surd (\sigma \Delta \pi /\pi_{c})$$

Measurements were repeated before and after perfusion with neuraminidase for 20–25 min, matching apparent solute permeability experiments described above.

### Determination of true diffusive solute permeability

Convective and diffusive fluxes of albumin contribute to the apparent solute permeability coefficient for albumin (*P*_sBSA_). The relative importance of these processes were discriminated using the recast form of the Patlak equation derived by Curry (Curry, 1984) and as subsequently expressed by Fu *et al*. (1998):

(5) Ps=PdPe(ePe−1)+Lp(1−σ)ΔP eff

where

(6) Pe=Lp(1−σ)ΔP eff Pd

and

(7) ΔP eff =ΔP−σ alb ΔΠ alb

where *P*_d_ represents the true solute diffusive permeability coefficient for albumin across the vascular wall, and *Pe* is the Peclet number (ratio of convective to diffusive flux).

### Statistical analysis

All statistics were calculated using Prism (GraphPad) software. Statistical significance was considered positive when a *P* value of < 0.05 had been demonstrated. Unless otherwise expressed, all data are presented as mean ± standard error of the mean (SEM). All *n* values represent the number of animals used unless otherwise stated. Two‐sided parametric tests were employed for analysis of true value estimates of glycocalyx parameters and *P*_sBSA_ *in vivo*, as well as glycocalyx depth measured from electron micrographs, and the following statistical tests were employed: Pearson's rank for examination of correlation, paired *t* test, two‐way ANOVA, and one‐way ANOVA were used for paired experiments; unpaired *t* tests were used for unpaired comparisons. All *post hoc* analyses used Bonferroni post‐tests

## Results

### Endothelial glycocalyx

**Figure 3:**
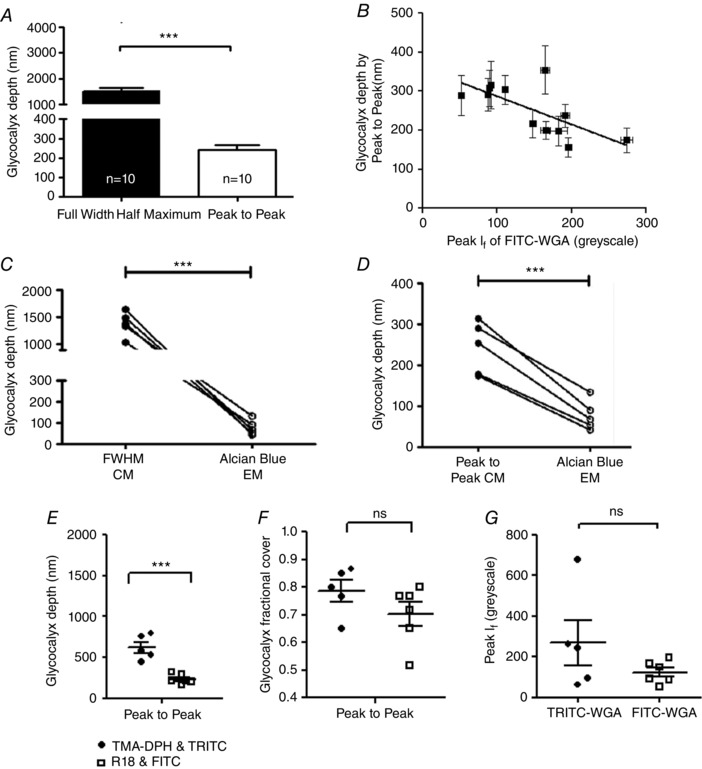
Endothelial glycocalyx depth in an individual vessel varies by more than an order of magnitude, according to the imaging and analysis method *A*, microvessels were imaged immediately after perfusion with FITC‐WGA (glycocalyx) and R18 (cell membrane). A line was drawn across the full diameter of the vessel and the depth of the endothelial glycocalyx estimated from: the full width measured at the point of half‐maximal fluorescence intensity of FITC‐WGA (FWHM); and the anatomical distance between peak signal from R18 (cell membrane) and FITC‐WGA (sialic acids within endothelial glycocalyx) (peak to peak). FWHM analysis generated significantly higher values of glycocalyx depth than peak to peak analysis (^***^ *P* < 0.001 paired *t* test). *B*, the depth of the glycocalyx (peak to peak method) (*y*‐axis) was inversely related to the peak fluorescence intensity (*I*_f_) of FITC‐WGA (*x*‐axis) (*r* = −0.50, *P* < 0.05 Pearson's correlation). *C*, full‐width half‐maximum (FWHM) measurements of endothelial glycocalyx depth made from confocal microscopy (CM) images were significantly greater than subsequent depth measurements of Alcian Blue‐labelled endothelial glycocalyx imaged with electron microscopy (EM) in the same vessel (^***^ *P* < 0.001 paired *t* test). *D*, peak to peak measurements of endothelial glycocalyx depth from confocal microscopy (CM) images were also significantly greater than measurements of Alcian Blue‐labelled endothelial glycocalyx depth measurements from electron microscopy (EM) in the same vessel (^***^ *P* < 0.001 paired *t* test). *E*, glycocalyx depth measurements made in TMA‐DPH and TRITC‐WGA‐labelled vessels by the peak to peak method are significantly greater than peak‐to‐peak‐determined endothelial glycocalyx depth measurements made in R18 and FITC‐WGA‐labelled vessels (*n* = 6) (^***^ *P* < 0.001 unpaired *t* test). There is no significant difference between fractional glycocalyx cover (*F*) or peak WGA fluorescence intensity (*I*_f_) (*G*) in TRITC‐WGA‐labelled vessels and FITC‐WGA‐labelled vessels (all non‐significant (ns), *P* > 0.05 unpaired *t* test).

Estimates of endothelial glycocalyx depth made in vessels perfused with R18 and FITC‐WGA using the FWHM method yielded significantly higher measurements (1507 ± 136.9 nm, *n* = 10) than those determined in the same vessels using the peak to peak method of analysis (243.6 ± 22.44 nm, *n* = 10) (*P* < 0.001, paired *t* test; Fig. 3 *A*). Using peak to peak measurements the fraction of vessel wall yielding positively resolvable glycocalyx measurements was 0.77 ± 0.04 (*n* = 10) and the mean peak *I*_f_ of FITC‐WGA across vessel walls was 176.4 ± 21.17 (*n* = 10). As the peak *I*_f_ of the FITC‐WGA signal increased, the paired peak to peak depth of endothelial glycocalyx significantly decreased, indicating that the peak *I*_f_ of FITC‐WGA may be influencing the anatomical location of the peak *I*_f_ of R18, thereby resulting in a reduction in peak to peak endothelial glycocalyx depth estimates (*P* < 0.05 by Pearson's correlation, *r* = −0.6975; Fig. 3 *B*).

Endothelial glycocalyx depth measured *in vivo* using both analysis methods (peak to peak and FWHM) were compared with the depth of endothelial glycocalyx measured at the same anatomical site of the same vessel *ex vivo* from paired electron micrographs. Paired comparisons demonstrated that endothelial glycocalyx depth estimated with FWHM (1376 ± 99.77 nm, *n* = 5) (Fig. 3 *C*) and peak to peak (243.6 ± 22.44, *n* = 5) (Fig. 3 *D*) techniques both yielded significantly greater estimates of depth than that estimated from paired electron micrographs (78.09 ± 16.16 nm, *n* = 15) (*P* < 0.001, paired *t* test). Endothelial glycocalyx depth measurements were therefore 3.1‐ to 17.6‐fold greater using confocal microscopy *in vivo* than measurements at the same site using electron microscopy *ex vivo*, with the broad range dependent on the method used to analyse the confocal microscopy images.

Measurements of glycocalyx depth for TRITC‐WGA and TMA‐DPH peak to peak (622.7 ± 67.18 nm *n* = 5) methods were, on average, significantly greater than the previous depth estimates for FITC‐WGA and R18 peak to peak (231.4 ± 24.4 nm, *n* = 6, *P* < 0.001 by unpaired *t* test) (Fig. 3 *E*). Fractional coverage by resolvable endothelial glycocalyx was not significantly different using the two sets of fluorescent labels (TMA‐DPH, TRITC‐WGA: 0.7867 ± 0.03851, *n* = 5; R18, FITC‐WGA: 0.7028 ± 0.04291, *n* = 6) (*P* > 0.05 unpaired *t* test) (Fig. 3 *F*). Peak fluorescence intensity measurements were also not significantly different between TRITC‐WGA (269.0 ± 109.5 greyscale units *n* = 5) and FITC‐WGA (123.6 ± 22.30 *n* = 6 greyscale units) (*P* > 0.05 unpaired *t* test) (Fig. 3 *G*). Unlike the significant relation between endothelial glycocalyx depth measured with the peak to peak method and the peak *I*_f_ of FITC‐WGA signal, there was no significant relation between peak to peak determined glycocalyx depth and the peak *I*_f_ of TRITC‐WGA signal (*r* = 0.29, *P* > 0.5 Pearson's correlation).

### Apparent solute permeability

**Figure 4:**
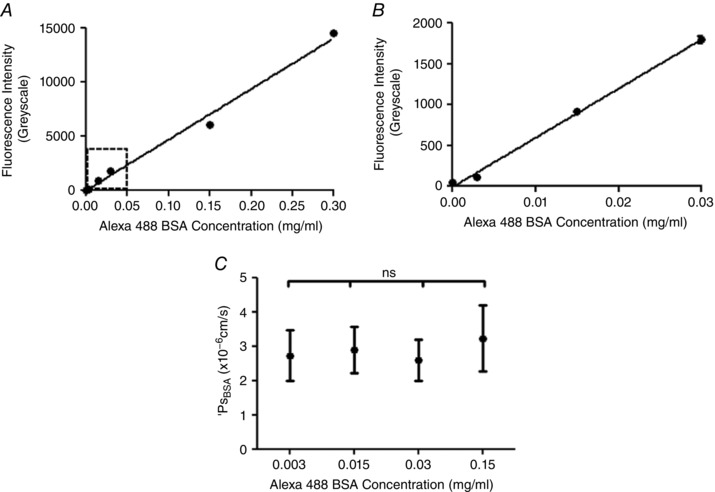
Imaging and analysis of solute permeability *in vivo* *A*, the mean fluorescence intensity of Alexa Fluor 488‐conjugated BSA (*I*_f_: *y*‐axis) was measured and quantified within a cannulated and perfused microvessel *in vivo*, and plotted against the concentration of Alexa Fluor 488‐conjugated BSA being perfused (*x*‐axis). The measured *I*_f_ is linearly related to the concentration of Alexa Fluor 488‐conjugated BSA being perfused (*r* ^2^ = 0.9916, *P* < 0.0001 Pearson's correlation). *B*, an expanded plot of the lower range of *I*_f_ *vs*. Alexa Fluor 488‐conjugated BSA concentration data, demonstrating that this linear relation holds at the range of low concentrations of Alexa Fluor 488‐conjugated BSA used in solute permeability experiments (0.03 mg ml^−1^) *C*, *P*_sBSA_ was calculated in the same perfused microvessel *in vivo* perfused with different concentrations of Alexa Fluor 488‐conjugated BSA. There was no significant difference between the *P*_sBSA_ values calculated from the *I*_f_ for each of these perfusates (ns, *P* = 0.9404, one‐way ANOVA).

To test the assumption of a linear relationship between the number of Alexa 488‐BSA molecules in a perfused vessel with the mean *I*_f_ measured in that same vessel *in vivo*, individual vessels were imaged during consecutive perfusion with the following concentrations of Alexa 488 BSA: 0, 0.003, 0.015, 0.03, 0.15 and 0.3 mg ml^−1^ of Alexa 488 BSA. There was a significant linear relationship between the number of Alexa Fluor 488 BSA molecules and the mean *I*_f_ measured within the perfused vessel (*r* ^2^ = 0.9916, *P* < 0.0001, Pearson's correlation) (Fig. 4 *A* and *B*). There was no significant difference between *P*_sBSA_ estimates made using these different concentrations of Alexa 488 BSA in the same microvessel (*P* > 0.05 by one‐way ANOVA) (Fig. 4 *C*).

### Importance of endothelial glycocalyx sialic acids

**Figure 5:**
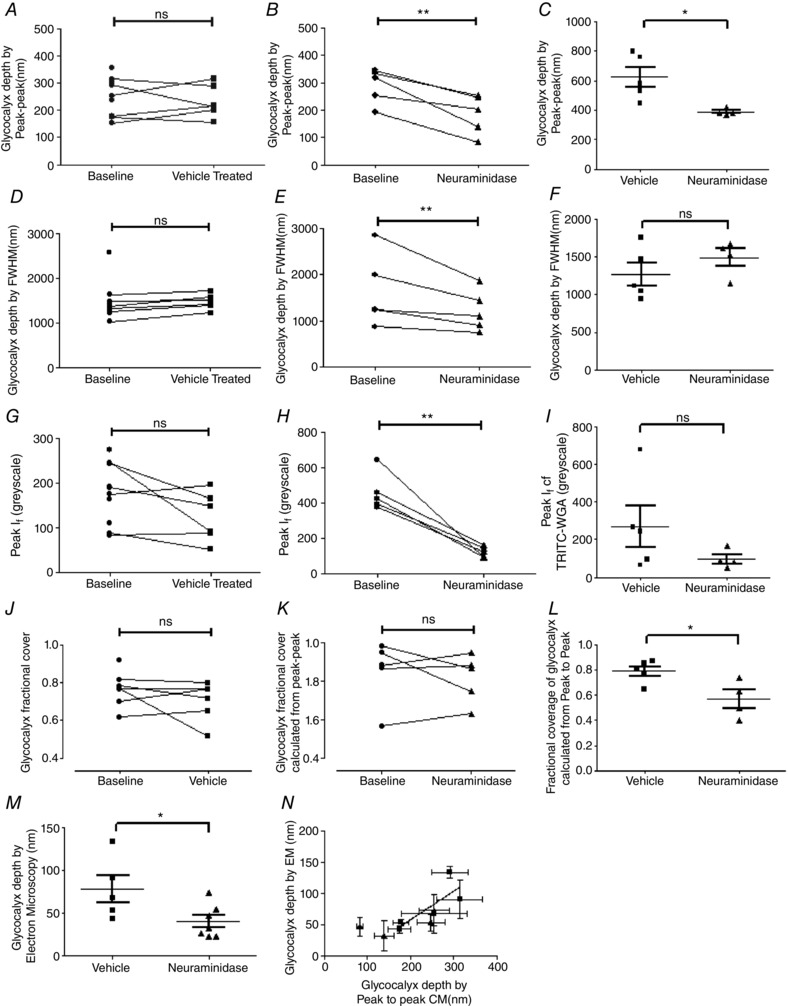
Disruption of sialic acids reduces endothelial glycocalyx depth and coverage Endothelial glycocalyx parameters were determined in vessels with sialic acid disruption achieved by 20 min perfusion with 40 mg ml^−1^ BSA supplemented with 2 U ml^−1^ neuraminidase (filled triangles), or in control vessels perfused with 40 mg ml^−1^ BSA vehicle alone (baseline: filled circles; vehicle perfusion; filled squares). Glycocalyx depth determined using the peak to peak method of analysis was no different before and after perfusion with vehicle (*A*), but was significantly reduced after neuramindase perfusion (*B*), (ns: *P* > 0.05; ^**^ *P* < 0.01, two‐way ANOVA with Bonferroni *post hoc* analysis). This neuraminidase‐induced reduction in peak‐to‐peak‐determined measurements of endothelial glycocalyx depth was reproduced in unpaired experiments (*C*), in which endothelial glycocalyx depth was determined once only, after earlier measurements of solute permeability in the same vessel during perfusion with either 40 mg ml^−1^ BSA vehicle alone (filled squares) or 40 mg ml^−1^ BSA supplemented with 2 U ml^−1^ neuraminidase (filled triangles) (^*^ *P* < 0.05, unpaired *t* test). Likewise, glycocalyx depth determined with the full‐width half‐maximum (FWHM) method was unaltered by perfusion with vehicle solution (*D*), but was significantly reduced after neuraminidase perfusion (*E*) (ns: *P* > 0.05; ^**^ *P* < 0.01, two‐way ANOVA with Bonferroni *post hoc* analysis). However, in separate measurements in which endothelial glycocalyx depth was measured only once by the FWHM after initial solute permeability measurements during perfusion with either 40 mg ml^−1^ BSA vehicle alone (filled squares) or 40 mg ml^−1^ BSA supplemented with 2 U ml^−1^ neuraminidase (filled triangles), no significant difference was determined (ns, *P* > 0.05, unpaired *t* test) (*F*). Identical results (as for FWHM) were also obtained when determining the peak *I*_f_ of WGA‐labelled endothelial glycocalyx in paired (*G* and *H*; ns: *P* > 0.05; ^**^ *P* < 0.01, two‐way ANOVA with Bonferroni *post hoc* analysis) and unpaired (*I*; ns, *P* > 0.05, unpaired *t* test) experiments. The fractional coverage of the vessel wall with endothelial glycocalyx resolvable with the peak to peak method of analysis was no different before and after perfusion with either vehicle or neuraminidase (*J* and *K*; ns: *P* > 0.05, two‐way ANOVA with Bonferroni *post hoc* analysis), but a significant reduction was observed after neuraminidase perfusion in unpaired experiments (*L*; ^*^ *P* < 0.05, unpaired *t* test). Endothelial glycocalyx depth measured in electron micrographs was significantly lower in neuramindase‐perfused vessels, as compared with vehicle‐perfused vessels (^*^ *P* < 0.05, unpaired *t* test) (*M*). There was a significant positive correlation between the depth of endothelial glycocalyx determined in electron microscopy (EM) images with the depth of endothelial glycocalyx determined by the peak to peak analysis method in confocal microscopy (CM) images in the same vessel (*N*; *r* = 0.75, *P* < 0.05 Pearson correlation).

To determine whether endothelial glycocalyx sialic acids contribute to endothelial glycocalyx structure and vessel wall permeability, endothelial glycocalyx parameters and solute permeability were measured in individual vessels before and after (or with and without) disruption of terminal sialic acids by perfusion with neuraminidase. With the peak to peak method of analysis, there was no significant difference between glycocalyx depth at baseline (243.6 ± 22.44 nm, *n* = 10) and that measured after 20 min of treatment with vehicle (231.4 ± 24.4 nm, *n* = 6) (*P* > 0.05, two‐way ANOVA Bonferroni *post hoc* analysis) (Fig. 5 *A*), but glycocalyx depth was significantly reduced within single vessels after 20 min of treatment with neuraminidase (185.0 ± 32.78 nm) when compared to baseline (288.9 ± 28.74 nm) (*P* < 0.01, *n* = 5, two‐way ANOVA Bonferroni *post hoc* analysis) (Fig. 5 *B*). The technique with the peak to peak method of analysis was sufficiently sensitive to detect changes in endothelial glycocalyx depth in unpaired comparisons of vehicle‐ or neuraminidase‐perfused vessels labelled with TRITC‐WGA and TMA‐DPH (rather than FITC‐WGA and R18, as described above) (vehicle treatment: 622.7 ± 67.18 nm; neuraminidase treatment: 386.3 ± 10.82 nm; *P* < 0.05, unpaired *t* test) (Fig. 5 *C*).

Removal of sialic acid residues with 20 min of treatment with neuraminidase also reduced the depth of endothelial glycocalyx as determined by the FWHM method of analysis (1646 ± 352.6 nm, *n* = 5, at baseline; 1223 ± 199.4 nm, *n* = 5, after neuraminidase) (*P* < 0.05, *n* = 5, two‐way ANOVA Bonferroni *post hoc* analysis), with no significant change in endothelial glycocalyx depth as determined by the FWHM method when vessels were treated with vehicle (1479 ± 68.81 nm) compared to baseline (1355 ± 84.03 nm) (*P* > 0.05, two‐way ANOVA Bonferroni *post hoc* analysis) (Fig. 5 *D* and *E*). Unpaired comparisons (TRITC‐WGA and TMA‐DPH) of the FWHM measure of glycocalyx depth did not have sufficient discrimination to demonstrate a significant reduction in glycocalyx depth following treatment with neuraminidase (1492 ± 116.8 nm) when compared to vehicle treated control (1265 ± 150.5 nm) (*P* > 0.05 by unpaired *t* test) (Fig. 5 *F*).

The fluorescence intensity of FITC‐WGA (*I*_f_) was not significantly altered after perfusion for 20 min with vehicle (123.6 ± 22.30, *n* = 6) when compared to that measured at baseline (176.4 ± 21.17, *n* = 10) (*P* > 0.05, two‐way ANOVA Bonferroni *post hoc* analysis) (Fig. 5 *G*), but was significantly reduced following 20 min of neuraminidase perfusion (124.9 ± 13.53) when compared to that imaged at baseline (460.9 ± 48.21) (*n* = 5, *P* < 0.001, two‐way ANOVA Bonferroni *post hoc* analysis) (Fig. 5 *H*). Unpaired comparisons of peak *I*_f_ of TRITC‐WGA did not have sufficient discrimination to demonstrate a statistically significant change to glycocalyx staining intensity following treatment with neuraminidase (95.63 ± 24.53 nm) when compared to the vehicle‐treated control (269.0 ± 109.5 nm) (*P* > 0.05 by unpaired *t* test) (Fig. 5 *I*).

The proportion of vessels with a glycocalyx depth greater than zero (fractional coverage), determined using the peak to peak method, was unchanged from baseline coverage (0.7650 ± 0.02563, *n* = 10) after perfusion of vehicle (0.7028 ± 0.04291, *n* = 6) (*P* > 0.05, two‐way ANOVA Bonferroni *post hoc* analysis), and likewise was unchanged before (0.8500 ± 0.07397, *n* = 5) and after perfusion with neuraminidase (0.8167 ± 0.05603, *n* = 5) (*P* > 0.05, two‐way ANOVA Bonferroni *post hoc* analysis) (Fig. 5 *J* and *K*). Unpaired comparisons (using TRITC‐WGA and TMA‐DPH) did reveal a significant reduction in glycocalyx fractional coverage following neuraminidase treatment (0.57 ± 0.07) when compared to the vehicle‐treated control (0.79 ± 0.04) (*P* < 0.05 by unpaired *t* test) (Fig. 5 *L*).

Measurement of endothelial glycocalyx depth within electron micrographs revealed a significant reduction in glycocalyx depth for vessels treated with neuraminidase (40.01 ± 7.317 nm, *n* = 7) when compared to vessels treated with vehicle alone (78.09 ± 16.16 nm, *n* = 5) (*P* < 0.05, by unpaired *t* test) (Fig. 5 *M*). The depth of the endothelial glycocalyx determined *in vivo* from peak to peak analysis demonstrated a significant positive linear relationship to the depth estimates of the endothelial glycocalyx in the same vessels using electron micrographs (*r* = 0.7515, *P* < 0.05, Pearson's correlation, 9 pairs) (Fig. 5 *N*). However, none of the other measurements of endothelial glycocalyx parameters obtained from confocal microscopy images (peak *I*_f_, fractional coverage) correlated with the endothelial glycocalyx depth measurements from electron microscopy images (FWHM: *r* = 0.1362, *n* = 9 pairs, *P* > 0.05 Pearson's correlation; peak *I*_f_: *r* = −0.5184, *n* = 9 pairs, *P* > 0.05 Pearson's correlation; fractional coverage: *r* = −0.2734, *n* = 9 pairs, *P* > 0.05 Pearson's correlation).

**Figure 6:**
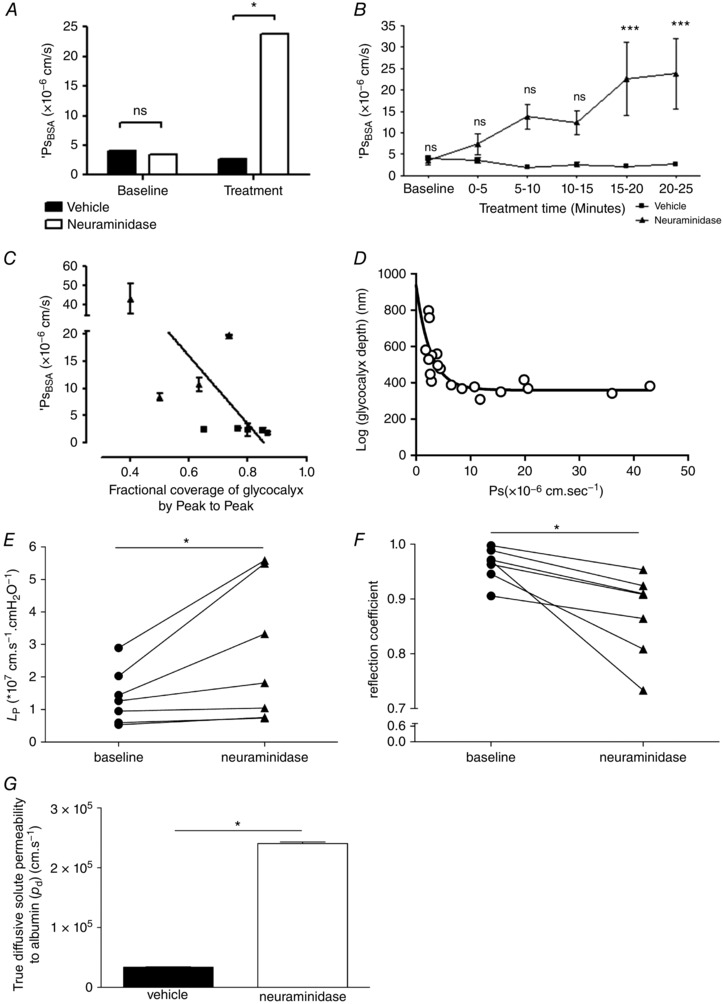
Disruption of sialic acids increases microvascular permeability to water and albumin *A*, apparent solute permeability to albumin (*P*_sBSA_) was measured in microvessels before (baseline) and 20–25 min after (treatment) perfusion with either vehicle (filled bars) or neuraminidase (open bars). Sialic acid disruption with neuraminidase perfusion significantly increased *P*_sBSA_ (^*^ *P* < 0.05, two‐way ANOVA *post hoc* Bonferroni analysis), with no significant change in *P*_sBSA_ in vehicle‐perfused control vessels (ns, *P* > 0.05, two‐way ANOVA *post hoc* Bonferroni analysis). *B*, *P*_sBSA_ increased progressively over time during neuraminidase perfusion (filled triangles), but remained unaltered during vehicle perfusion (filled squares). A significant increase in *P*_sBSA_ was noted after 15–20 min treatment with neuraminidase, matching the time course of neuraminidase‐induced endothelial glycocalyx disruption (^***^ *P* < 0.001, two‐way ANOVA, Bonferroni *post hoc* analysis). A significant inverse correlation was noted between *P*_sBSA_ and fractional coverage by resolvable endothelial glycocalyx determined with the peak to peak method (*C*) (*r* = −0.73, *P* < 0.05 Pearson correlation). A significant relation between *P*_sBSA_ and endothelial glycocalyx depth determined with the peak to peak method was best described by a single one‐phase decay curve with a relationship of depth = 330 nm + 607 nm × e^−0.38×^ *^P^* ^s^ (*D*). Microvessel hydraulic conductivity (*L*_P_) was significantly increased after sialic acid disruption with 20–25 min perfusion with neuraminidase (filled triangles) compared with baseline measurements in the same vessel (filled circles) (^*^ *P* < 0.05, Wilcoxon) (*E*). Microvessel reflection coefficient to albumin was significantly reduced after sialic acid disruption with 20–25 min perfusion with neuraminidase (filled triangles) compared with baseline measurements in the same vessel (filled circles) (^*^ *P* < 0.05, Wilcoxon) (*F*). There was a significant increase in true diffusive solute permeability to albumin after sialic acid disruption with neuraminidase (^*^ *P* < 0.001, unpaired *t* test) (*G*).

Treatment of perfused microvessels with vehicle solution for 20–25 min did not significantly alter *P*_sBSA_ (from (4.0 ± 0.43) × 10^−6^ cm s^−1^ to (2.6 ± 0.44) × 10^−6^ cm s^−1^; *n* = 5 pairs; *P* > 0.05, two‐way ANOVA with Bonferroni's correction), but disruption of sialic acids with neuraminidase treatment did significantly increase *P*_sBSA_ 6.8‐fold (from (3.5 ± 1.0) × 10^−6^ cm s^−1^ to (23.8 ± 8.3) × 10^−6^ cm s^−1^; *n* = 4 pairs; *P* < 0.001 by two‐way ANOVA) (Fig. 6 *A*). The rise in permeability in response to neuraminidase treatment was progressive and linear (*r* ^2^ = 0.9216; *P* < 0.01, Pearson's correlation) (Fig. 6 *B*). There was a significant inverse linear relationship between *P*_sBSA_ and endothelial glycocalyx fractional coverage in the same vessels in neuraminidase‐treated and vehicle‐treated vessels (*r* = −0.731, *n* = 9 pairs, *P* < 0.05 Pearson's correlation) (Fig. 6 *C*). Likewise there was an inverse relationship between *P*_sBSA_ and endothelial glycocalyx depth as assessed by the peak to peak analysis of TRITC‐WGA in the same vessels in neuraminidase‐treated and vehicle‐treated vessels, (*r* = −0.80, *P* < 0.05 by Spearman's correlation) but this relationship was not linear (best fit: single one‐phase decay curve; depth = 330 nm + 607 nm × e^−0.38×^ *^P^* ^s^) (Fig. 6 *D*).

Over the same time course, disruption of sialic acids with neuraminidase treatment also significantly increased hydraulic conductivity by 1.9‐fold (baseline: (1.39 ± 0.32) × 10^−7^ cm s^−1^ cmH_2_O^−1^; neuraminidase: (2.68 ± 0.81) × 10^−7^ cm s^−1^ cmH_2_O^−1^; *n* = 7 pairs; *P* < 0.05, Wilcoxon) (Fig. 6 *E*) and significantly reduced the reflection coefficient (baseline: 0.96 ± 0.01; neuraminidase: 0.87 ± 0.03; *n* = 7 pairs, *P* < 0.05, paired *t* test) (Fig. 6 *F*).

Using measured and calculated parameter values (*P*_sBSA_, *L*_p_, σ, π_BSA_) from these experiments, and calculating the Peclet number for these experiments, true diffusive BSA solute permeability coefficient values (*P*_d_) were 7‐fold and significantly increased by disruption of sialic acids by neuraminidase treatment (3.38 × 10^−6^ cm s^−1^ under baseline conditions; 2.41 × 10^−5^ cm s^−1^ after neuraminidase treatment; *P* < 0.001, unpaired *t* test) (Fig. 6 *G*).

## Discussion

In this study we have successfully developed methods for paired, real‐time, direct *in vivo* assessment of endothelial glycocalyx structure and dependent microvascular function (permeability). These techniques demonstrate that measurements of the depth of endothelial glycocalyx are highly dependent on the labelling, detection and analysis methods employed, and vary by more than an order of magnitude. Highest values are obtained by full‐width half‐maximum analysis of fluorescently labelled WGA‐lectin binding to the endothelial glycocalyx, and lowest values are obtained from electron micrographs of Alcian Blue‐labelled endothelial glycocalyx from the same vessel. Estimating the anatomical distance between peak signals from endothelial glycocalyx and endothelial cell membrane labels with distinct fluorescence emission spectra provides the most sensitive and reliable measure of endothelial glycocalyx depth. Sialic acid residues within the endothelial glycocalyx regulate glycocalyx structure, as well as the permeability of the vessel wall to both water and albumin. Neuraminidase‐induced changes in glycocalyx structure (by fluorescence and electron microscopy) correlated with changes in transvascular solute flux.

Sialic acids cap a range of glycoproteins within the endothelial glycocalyx (e.g. Varki, 2008) and are present at high density on the endothelial cell surface (<50 × 10^6^ μm^−2^, Born & Palinski, 1989, including in human microvessels, Tatsuzuki *et al*. 2009), and are potentially found at increased density at the endothelial cell surface adjacent to the dominant route for water and solute flux across the vessel wall: interendothelial clefts (Bai & Wang, 2014). The current studies demonstrate that disruption of sialic acid residues within the endothelial glycocalyx reduces the degree of reflection of albumin by the microvessel wall, and increases the conductance of water, and therefore sialic acid residues within the endothelial glycocalyx are principal regulators of microvessel permeability.

These findings are in direct conflict with the only other investigation of the effect of sialic acid disruption on true permeability coefficients *in vivo* (Mason *et al*. 1977), in which neuraminidase had no effect on mesenteric microvessel hydraulic conductivity. Whilst it is possible that species differences (frog *vs*. rat) account for this difference, the highly conserved nature of endothelial sialic acid expression (Aamelfot *et al*. 2014) argues against this possibility. We suggest that it is more likely that temperature differences (frog ∼15°C; rat 37°C) permit greater enzyme activity and hence greater degree of sialic acid disruption in the current study, thereby revealing an important role of sialic acids in regulating permeability. The previous study in frogs did not perform paired labelling of glycocalyx or other ultra‐structural components, and thus active changes in structure assumed to be brought about by neuraminidase were not confirmed (Mason *et al*. 1977) as they have been here.

Other evidence supporting the concept of sialic acids regulating microvascular permeability arise from systems in which the measures of permeability coefficients are confounded by the model used or by unregulated variables. For example, disruption of endothelial glycocalyx sialic acids with neuraminidase was shown to increase permeability and reduce glycocalyx depth of endothelial cells *in vitro* (Singh *et al*. 2007; Cioffi *et al*. 2012). Whilst cultured endothelial cells display permeability coefficients that are orders of magnitude greater than those observed in intact vessels *in vivo* (Curry, 2005), and possess an endothelial glycocalyx that differs substantially from that observed on intact vessels (Chappell *et al*. 2009), these findings are consistent with the demonstrated importance of sialic acids in regulating multiple permeability coefficients *in vivo* in the current report. Investigations into the effects of neuraminidase on water and albumin flux in other microvessel beds support our findings that true microvessel wall permeability coefficients are regulated by sialic acid residues. One such study observed alveolar oedema formation within isolated‐perfused lungs from rats treated with neuraminidase (0.5 U ml^−1^ for 30 min) demonstrating an ∼8‐fold increase in transvascular perfusate flux (calculated from the change in organ weight at different perfusion pressures) indicating that sialoglycoproteins expressed within the endothelial glycocalyx of pulmonary microvessel beds may influence solute and water flux (Cioffi *et al*. 2012). Since sialic acids also regulate glycocalyx‐mediated mechanotransduction (Tarbell & Pahakis, 2006) and consequent vessel dilatation (Kumagai *et al*. 2009), observed increases in solute or water flux following neuraminidase treatment (Cioffi *et al*. 2012; Salmon *et al*. 2012) may represent a vasodilatation‐induced increase in the hydrostatic forces driving solute and water flux rather than a true change in the permeability of the vessel wall. The current studies confirm that, over and above regulating vessel diameter and hence the magnitude of Starling forces driving solute and fluid flux, sialic acids also regulate the permeability of microvessel walls.

There are a number of biophysical and biochemical mechanisms through which sialic acids may regulate microvascular permeability. The contribution of the endothelial glycocalyx to hydraulic permeability is thought to be imparted largely through glycocalyx depth (Adamson, 1990), with a reduction in glycocalyx thickness sufficient to explain increased hydraulic conductivity, as observed in response to sialic acid disruption here. The endothelial glycocalyx has been calculated to contribute approximately 60% to the hydraulic resistance of the vessel wall (Adamson *et al*. 2004). The ∼2‐fold increase in hydraulic conductivity following neuraminidase treatment is comparable to the ∼2.5‐fold increase in hydraulic conductivity following pronase treatment of frog mesenteric microvessels (Adamson, 1990), and also similar to the ∼2.3‐fold increase in hydraulic conductivity observed in rats with spontaneous proteinuric chronic kidney disease and reduced glycocalyx depth (Salmon *et al*. 2012). The relationship between glycocalyx depth and albumin permeability has been less clear: the non‐linear relation between these parameters indicated here (Fig. 6 *D*) illustrates one possible aspect of that relationship. The presence of glycocalyx after neuraminidase treatment with a reduction in thickness of a similar relative level (60% for confocal, 50% for electron microscopy) suggests that the glycocalyx needs to reach a minimal depth before being able to impede albumin flux. Alternative explanations include a potential change in glycocalyx coverage in critical permeability‐determining regions, such as in microdomains overlying interendothelial clefts. Furthermore, since other structural and biological aspects of the glycocalyx (such as 3‐D ultrastructure and electrical charge) have not been assessed with these techniques, parameters other than depth and spatial distribution may also impact on the relationship between glycocalyx structure and albumin permeability. This could be further investigated with more information on the structure of the glycocalyx, such as that generated using complex staining techniques (Arkill *et al*. 2011) to be able to build testable models of how the glycocalyx regulates albumin permeability.

The observed reduction in endothelial glycocalyx thickness is unlikely, however, to explain the observed reduction in reflection coefficient. Molecules are reflected back into the vessel lumen during convective drag because the functional pore aperture, determined by the combination of steric and electrochemical pore properties, is too small to allow large solute molecules (such as BSA) to pass. The ordered lattice‐like structure of the endothelial glycocalyx imparts efficient molecular sieving properties (Arkill *et al*. 2011), with the arrangement, dimensions and charge of the individual fibres of the lattice all dictating the efficiency with which solutes are reflected back into the vessel lumen under conditions of convective flux (Hu *et al*. 2000; Squire *et al*. 2001; Weinbaum *et al*. 2003; Arkill *et al*. 2011). Membrane‐bound components of the endothelial glycocalyx display the necessary quasi‐ordered arrangement to contribute to this ordered lattice (Squire *et al*. 2001), raising the possibility that sialic acid‐capped, membrane‐bound glycoproteins and/or proteoglycans may be important in regulating the molecular sieving and hence the reflection coefficient of microvessel walls. For example, sialic acid‐depleted glycoproteins may form a pore that provides less resistance to the passage of albumin molecules because of reduced net negative charge on the fibres surrounding the pore, thereby facilitating the passage of negatively charged albumin molecules through the pore. However, removal of sialic acid residues did not significantly alter the surface charge of endothelial cells (Vargas *et al*. 1989), and hence the neuraminidase effect observed here may be steric rather than electrochemical, through altering the shape, size or interactions between sialic acid capped molecules that comprise the pore. Combining the current structure–function studies with glycan‐specific electron dense labels (e.g. gold particle‐labelled lectins, Baldwin & Winlove, 1984) and Fourier transformation analysis to investigate changes to the ordered arrangement of sialic acid residues within the endothelial glycocalyx may be informative in this regard.

Sialic acid disruption might also (or alternatively) result in secondary changes within the endothelial glycocalyx, thereby altering permeability coefficients. For example, rearrangement or activation of glycocalyx‐embedded receptors or interendothelial cleft molecules such as VE‐cadherin may follow sialic acid disruption (Geyer *et al*. 1999). However, reorganisation of the interendothelial cleft molecules occurs hours after sialic acid disruption (Cioffi *et al*. 2012), more slowly than both the increase in permeability and the change in endothelial glycocalyx measurements observed over minutes in these studies. Direct modification of the endothelial glycocalyx therefore appears to be a more likely explanation for the changes in permeability measured here. Disrupting the interaction between sialoglycoprotein‐based arginine residues and albumin molecules may have reduced the adsorbed fraction of albumin within the endothelial glycocalyx, reducing albumin‐dependent sphingosine‐1‐phosphate signalling leading to active shedding of other glycocalyx (Adamson *et al*. 2012, 2014; Curry *et al*. 2012; Curry & Adamson, 2013; Zeng *et al*. 2013). However, these secondary changes again point to an important role for sialic acid residues within the endothelial glycocalyx in regulating microvessel permeability.

These simultaneous changes in microvessel permeability and endothelial glycocalyx structure have been revealed through novel techniques that permit examination of microvessel permeability coefficients as well as real‐time confocal microscopy and *post hoc* electron microscopy examinations of the endothelial glycocalyx covering the same portion of the same vessel. Neuraminidase‐induced changes in the endothelial glycocalyx structure identified with confocal microscopy correlated with parallel changes in the endothelial glycocalyx measured from electron micrographs of the same portion of the same vessel *in vivo*. A number of processing steps are required to visualise the endothelial glycocalyx by electron microscopy, including albumin‐free perfusion prior to Alcian Blue labelling, glutaraldehyde fixation and dehydration steps, each of which may affect glycocalyx appearance in electron micrographs. Nevertheless, the findings from the current report provide confidence that, at least under the conditions reported here, changes in the structure of the endothelial glycocalyx observed by standard electron microscopy techniques correlate well with both direct imaging of the endothelial glycocalyx *in vivo* (i.e. pre‐EM processing) and with the function of the vessel wall.

The absolute value of endothelial glycocalyx depth measurements with this method is within the range reported by others using a variety of techniques (for review see Curry & Adamson, 2012), but above the theoretical maximum threshold depth compatible with the demonstrated hydraulic conductivity of vessel walls (Curry & Adamson, 2013). Importantly, different analysis methods of the same lectin‐labelled images generated a 6‐fold range of glycocalyx depth measurements (FWHM to peak to peak methods), and the same analysis technique using different membrane and glycocalyx labels (peak to peak techniques) generated a ∼2.5‐fold range of glycocalyx depths. Even the lowest estimate of glycocalyx depth from confocal images was ∼3‐fold greater than depth assessed from electron micrographs. Clearly the method used to estimate glycocalyx depth has a large impact on the absolute value of endothelial glycocalyx depth reported, even when multiple assessments are made on the same vessel under identical conditions. This makes it difficult to compare studies using varied methodologies to judge whether there are true differences in the endothelial glycocalyx between different vessel beds. These findings suggest that quantifying relative changes in glycocalyx depth in response to individual experimental manipulations under identical experimental conditions may be more reliable than interpreting the absolute depth of the endothelial glycocalyx from varied techniques.

Nevertheless, the peak to peak method, based on the anatomical distance between the peak endothelial plasmalemmal signal and the peak glycocalyx‐bound lectin signal, (1) enabled discrimination of neuraminidase‐induced changes in confocal‐determined glycocalyx depth measurements under paired and unpaired conditions, (2) with different glycocalyx and membrane labels, (3) correlated well with changes in endothelial glycocalyx depth measured by electron microscopy, and (4) correlated with changes in vessel wall function, thereby proving the most sensitive and reliable real‐time *in vivo* method for quantifying changes in the endothelial glycocalyx. The choice of glycocalyx and endothelial cell membrane labels for these peak to peak assessments included FITC‐WGA/R18, with demonstrated limitations including fluorescence emission signal bleed through and interactions between the magnitude of fluorescence signal and glycocalyx depth. TRITC‐WGA/TMA‐DPH assessments did not suffer from either of these theoretical or measurement errors, but the variability of peak fluorescence signal was greater and will benefit from assessment in larger sample sizes. Peak to peak measurements were preferred over full‐width half‐maximum measurements, as previous studies have indicated the presence of lectin binding to sialic acid residues on the abluminal side of the endothelial cell (e.g.Oltean *et al*. 2015) that broaden the resulting fluorescence signal to generate falsely elevated estimates of ‘endothelial glycocalyx’ depth. Failing to discriminate between luminal and abluminal surface glycocalyx components may have contributed to some of the higher values for glycocalyx depth reported in the literature, particularly in the absence of simultaneous imaging of the endothelial cell surface (Yen *et al*. 2012). Full‐width half‐maximum, peak fluorescence intensity and fractional coverage analysis methods were not capable of discriminating neuraminidase‐induced changes in the endothelial glycocalyx reliably (e.g. in paired and unpaired studies); none correlated with changes by electron microscopy, and neither peak fluorescence intensity or full‐width half‐maximum measurements correlated with changes in solute permeability; indicating that comparative assessment of glycocalyx signal relative to the endothelial cell surface is an important facet of measuring glycocalyx depth.

These direct labelling methods for quantifying the endothelial glycocalyx are an important complement to indirect methods of glycocalyx assessment, in which the distance of a luminal tracer (electron‐dense lipid droplets, fluorescently labelled circulating molecules or microparticles, circulating cells) from the vessel wall is assumed to represent an impenetrable depth of endothelial glycocalyx. Many such methods need to take into account partial permeation of the tracer into the endothelial glycocalyx (Michel & Curry, 2009), as well as the influence of the glycocalyx on the spatial profile of substances flowing through the vessel (Pries *et al*. 1997). As such, changes in haemodynamics as well as changes in the endothelial glycocalyx could impact on the distance of the tracer from the vessel wall, thereby confounding the assumption that this distance represents the depth of the endothelial glycocalyx. Stop‐flow studies (Haraldsson, 2014) and studies on small diameter vessels (Damiano, 1998) have been employed to overcome this confounding influence. Direct glycocalyx labelling methods reported here complement the critical *in vivo* assessments of glycocalyx‐dependent vascular functions. These considerations are also important because of the recent spread of indirect methods of estimating endothelial glycocalyx depth into the clinical arena (Lee *et al*. 2014), using technologies that are not designed to directly measure the anatomical location of the vessel wall and therefore can only estimate a non‐perfused boundary region. Validation studies coupling these potentially revolutionary clinically applicable methodologies with direct assessment of the endothelial glycocalyx are warranted.

Our study has a number of potential limitations. Neuraminidase cleaves the *O*‐glycosidic linkages between the terminal neuraminic (sialic) acids and the subterminal sugars (Cassidy *et al*. 1965), but almost all enzymatic manipulations of endothelial glycocalyx components are prone to direct but off‐target enzymatic disruption of other components of the endothelial glycocalyx (Tarbell, 2010). Neuraminidase removes <5% of other components of the endothelial glycocalyx (Pahakis *et al*. 2007). Wheat germ agglutinin lectin also labels *N*‐acetyl‐glucosamine residues within various glycosaminoglycans, although more than 60% of WGA‐labelled structures are sialic acids (Barker *et al*. 2004). Overall, the >95% specificity of neuraminidase and >60% specificity of WGA for sialic acid residues indicates that no more than 2% of the observed reduction in WGA signal after neuraminidase treatment is likely to be due to an effect on other glycocalyx structures.

Three parameters of microvascular permeability were measured in rat mesenteric microvessels under identical experimental conditions before and after exposure to neuraminidase, but true permeability coefficients (hydraulic conductivity and reflection coefficient) were measured in different vessels from those in which measurements of endothelial glycocalyx were made. This represents a technical limitation, since different microscopes were required for confocal and light microscopy studies, and it was not practical to maintain anaesthesia and transport animals between rigs. Nevertheless there is good concordance amongst the results, and changes in apparent solute permeability in one set of experiments are explained by the observed changes in hydraulic conductivity and reflection coefficient observed in parallel experiments. Of note, however, is that despite this concordance the magnitude of the *P*_sBSA_ values reported here (∼2.8 × 10^−6^ cm s^−1^ under normal and vehicle‐treated conditions) is greater than the ∼0.4 to 1.0 × 10^−6^ cm s^−1^ measured in rat mesenteric microvessels by other researchers (Fu & Shen, 2004; Cai *et al*. 2012; Curry *et al*. 2012; Adamson *et al*. 2014). In contrast, hydraulic conductivity values (1.39 × 10^−7^ cm s^−1^ cmH_2_O^−1^) are in the range reported previously (1.2–2.43 × 10^−7^ cm s^−1^ cmH_2_O^−1^; Kendall & Michel, 1995; Michel & Kendall, 1997; Curry *et al*. 2003, 2012; Salmon *et al*. 2009) as are reflection coefficient values (0.96; e.g. 0.94, Kendall & Michel, 1995). This suggests that technical factors relating to the *P*_sBSA_ experiments may be responsible for these higher *P*_sBSA_ values, such as reduced S1P content of erythrocyte‐free perfusate in *P*_sBSA_ and glycocalyx imaging studies (necessitated by the use of narrow‐lumen double‐barrelled theta pipettes in contrast to erythrocyte‐containing perfusate in hydraulic conductivity/reflection coefficient studies; Curry *et al*. 2012), or an influence of the Alexa 488 label applied to BSA (as demonstrated for other fluorescent labels; Bingaman *et al*. 2003). Low S1P conditions may compromise glycocalyx and intercellular junction integrity, affecting baseline permeability states, and this hinders detailed biophysical interpretation of sialic acid‐regulated pathways for transvascular passage of albumin.

The perfused single vessel methodology adopted here has also been critiqued in the literature. However, single vessel perfusion permeability techniques have been described as the ‘squid giant axon’ of permeability studies (Curry, 2005); the current report extends their utility to real‐time *in vivo* structure–function assessments of the endothelial glycocalyx as a complement to the range of other microvascular endothelial glycocalyx studies already available or in development.

Given the widespread importance of the endothelial glycocalyx for normal physiology and a broad number of disease states, we have expanded the range of techniques that permit direct studies of the structure of the endothelial glycocalyx, as well as dependent microvascular functions. Through these novel techniques, we demonstrate that sialic acid residues within the endothelial glycocalyx are critical regulators of microvascular permeability to both water and albumin.

## Additional information

### Competing interests

None declared.

### Author contributions

All authors interpreted the data and revised the article critically for important intellectual content. K.P.A., C.R.N. and D.O.B. also analysed data. K.B.B., K.P.A., C.R.N., D.O.B. and A.H.J.S. designed the experiments. K.B.B. and A.H.J.S. collected and analysed the data at the University of Bristol, and drafted the article. A.H.J.S. conceived the study. All authors have approved the final version of the manuscript and agree to be accountable for all aspects of the work. All persons designated as authors qualify for authorship, and all those who qualify for authorship are listed.

### Funding

The authors gratefully acknowledge funding from the Medical Research Council (G0802829) to A.S.
